# Virtual reality training of lucid dreaming

**DOI:** 10.1098/rstb.2019.0697

**Published:** 2020-12-14

**Authors:** Jarrod Gott, Leonore Bovy, Emma Peters, Sofia Tzioridou, Stefano Meo, Çağatay Demirel, Mahdad Jafarzadeh Esfahani, Pedro Reis Oliveira, Thomas Houweling, Alessandro Orticoni, Anke Rademaker, Diede Booltink, Rathiga Varatheeswaran, Carmen van Hooijdonk, Mahmoud Chaabou, Anastasia Mangiaruga, Erik van den Berge, Frederik D. Weber, Simone Ritter, Martin Dresler

**Affiliations:** 1 Donders Institute for Brain, Cognition and Behaviour, Radboud University Medical Centre, Nijmegen, The Netherlands; 2 Department of Psychology, Philipps University, Marburg, Germany; 3 Department of Psychology, University of Zurich, Zurich, Switzerland; 4 IRCCS San Raffaele Pisana, Rome, Italy; 5 Leibniz Institute for Resilience Research, Mainz, Germany; 6 School for Mental Health and Neuroscience, Maastricht University, Maastricht, The Netherlands; 7 Rivierduinen Institute for Mental Healthcare, Leiden, The Netherlands; 8 Technical University Berlin, Germany; 9 Department of Medical and Surgical Sciences (DIMEC), University of Bologna, Bologna, Italy; 10 Institute for Management Research, Radboud University, Nijmegen, The Netherlands

**Keywords:** lucid dreaming, virtual reality, metacognition, consciousness, REM sleep, dissociation

## Abstract

Metacognitive reflections on one's current state of mind are largely absent during dreaming. Lucid dreaming as the exception to this rule is a rare phenomenon; however, its occurrence can be facilitated through cognitive training. A central idea of respective training strategies is to regularly question one's phenomenal experience: is the currently experienced world *real*, or just a dream? Here, we tested if such lucid dreaming training can be enhanced with dream-like virtual reality (VR): over the course of four weeks, volunteers underwent lucid dreaming training in VR scenarios comprising dream-like elements, classical lucid dreaming training or no training. We found that VR-assisted training led to significantly stronger increases in lucid dreaming compared to the no-training condition. Eye signal-verified lucid dreams during polysomnography supported behavioural results. We discuss the potential mechanisms underlying these findings, in particular the role of synthetic dream-like experiences, incorporation of VR content in dream imagery serving as memory cues, and extended dissociative effects of VR session on subsequent experiences that might amplify lucid dreaming training during wakefulness.

This article is part of the theme issue ‘Offline perception: voluntary and spontaneous perceptual experiences without matching external stimulation'.

## 1. Introduction

Lucid dreaming is a state of consciousness during sleep whereby neurophysiologically demodulated aspects of cognition—self-reflection, critical analysis and introspective insight—are aberrantly restored and made available within one's dreams [1]. To the extent that the brain is capable of generating its own sensory content (divorced of external stimuli), lucid dreaming comprises a delicate centrepoint in a neurobiological balance: retaining wake-like levels of reflection and volitional control [2], but vastly surpassing waking imagery in its immersion and depth, while circumventing the myriad of hazards associated with psychopathological and pharmacological hallucination. Lucid dreaming therefore holds the mantle of being the closest thing the brain has—in terms of safe, endogenous capability—to experience fully immersive, authentic and convincing virtual reality (VR), delivering aesthetic and emotionally salient content that can approach waking life in its authenticity.

Lucid dreaming occurs relatively rarely under normal conditions; however, its frequency can be increased by different strategies. Classical lucid dreaming training involves critically questioning one's reality, particularly in dream-like or surreal situations [3,4]. Frequently having the waking thought ‘am I dreaming?' may then become a habituated cognitive process and be randomly reactivated while dreaming. Somewhat paradoxically, such reactivation can result in profound state changes at both the psychological and physiological level; merely dreaming that one ‘critically questions reality' can indeed result in that critique being performed authentically. Since volitional motor activity performed within one's dreams can recruit the same cortical regions as when waking [5,6], it stands to reason that volitional thought processes performed within one's dreams may also be capable of operating by this logic. In other words, reflecting upon the authenticity of one's environment while asleep can produce the necessary state changes in neurological activity as to generate lucidity as an explicit psychological endpoint. Since one of the defining hallmarks of rapid eye movement (REM) cognition is the overt inhibition of metacognitive processes [7], critically questioning one's waking reality serves as an overt mnemonic strategy for disrupting the typical inhibition of metacognitive processes within this state.

Performing critical evaluations of one's waking reality is not without its setbacks. Only genuine critiques would accordingly recruit sufficient cognitive processes as to generate lucidity once reactivated within a dream. Since waking life rarely contains dream-like or surreal moments, the provision of these environments via VR would be one way to increase the availability of convincingly dream-like experiences; within which a moment for genuine critical reflection is made available. Thus, the traditional method for lucid dream induction—critical questions about one's current state of mind and ‘reality checks'—may be greatly enhanced through the additional provision of VR environments with dream-like aesthetic properties. In this study, we administrated VR training to participants with limited to no prior VR experience and assessed whether this produced superior gains in dream lucidity against two control groups: an active control group which performed cognitive training only and a passive control group without any training. Our hypothesis was that VR-enhanced lucid dreaming training would be more effective in increasing lucid dreaming than cognitive training only, which in turn would be more effective than no training.

## 2. Methods

### (a) Participants and procedures

Forty-two participants were recruited from the campus of Radboud University in Nijmegen, The Netherlands. Participants were required to remember their dreams at least 3 days per week. Exclusion criteria constituted the presence of health or sleep-related issues, prescription of psychopharmacological medication, drug use exceeding recreational and legal standards, ongoing shift work and having more than one hour of previous VR experience. Three participants were excluded after data acquisition: one for having a ‘starting lucidity' that exceeded three standard deviations from the mean of the group; one for having a dream recall that fell under 50% (13/42 days with recalled dreams) and another for poor compliance and/or potential confabulation of behavioural results—specifically, dream reports that did not reflect daily questionnaire scores, and vice versa. This brought the final cohort size down to 39, with 13 in each group (for demographics, table 1). All participants signed informed consent in accordance with the approval of the local ethics committee.

**Table 1.** Demographics, questionnaire results and eye signal verification, given as means ± standard error or absolute numbers, respectively. MADRE = Mannheim dream questionnaire. DLQ = dream lucidity questionnaire; ‘baseline' = average of first 7 days, ‘final' = average of final 7 days. LuCiD = lucidity and consciousness in dreams scale. Lucidity verbally reported during the baseline/final week. LRLR = eye signal verification of lucid dreaming during polysomnography, reported and electrooculography (EOG)-verified by raters blind to the study condition.

	passive control	active control	virtual reality
n	13 (8 f/5 m)	13 (10 f/3 m)	13 (11 f/2 m)
age	24.8 ± 1.5	22.5 ± 0.9	22.0 ± 0.8
MADRE lucidity screening	1.15 ± 0.32	1.39 ± 0.35	1.31 ± 0.31
DLQ lucidity baseline week	2.9 ± 0.9	3.0 ± 0.7	3.4 ± 0.8
DLQ lucidity final week	2.0 ± 0.8	4.5 ± 1.2	6.3 ± 1.5
LuCiD insight baseline	5.2 ± 1.7	5.3 ± 1.7	3.8 ± 1.4
LuCiD insight final	3.6 ± 1.4	4.6 ± 0.7	8.7 ± 2.0
lucidity reported baseline/final	0 / 0	1 / 4	0 / 12
LRLR reported baseline/final	0 / 0	0 / 2	0 / 5
LRLR verified baseline/final	0 / 0	0 / 0	0 / 3

Participants were pseudo-randomized into three groups, comprising a VR group, in addition to *active* (AC) and *passive* (PC) control groups. Groups were stratified primarily by lucid dream frequency at screening according to the Mannheim dream questionnaire [8] and secondarily by gender and age.

All groups were required to keep a daily dream diary with instructions to write down the contents of their dream upon awakening and rate the lucidity experienced in their dreams using the dream lucidity questionnare (DLQ; [9]). Participants preceded this report by completing an ‘evening' section on the previous night, containing information that could potentially affect their ensuing sleep and dream experiences. This included questions about whether the participant had taken a nap during the previous day, their mood before going to sleep, how much alcohol they consumed, and their intended sleep time. On awakening, the dream would be hand written, and the questionnaire filled out, in addition to recording their wake time and mood upon awakening. Participants of the VR group further had to indicate if their dream content resembled the VR scenarios they were exposed to. All groups produced six weeks (42 days) of dream diary. One night in the first week of the study and three consecutive nights in the last week were recorded with home-based polysomnography (SOMNOscreen, SOMNOmedics, Germany). After each polysomnography, participants filled out the LuCiD questionnaire [10] in addition to the DLQ. All participants were instructed to verify any lucid dreams during polysomnography with eye signal verification [11], i.e. moving their eyes left-right-left-right if they realized they were dreaming. In the first week, in the final week, and in a follow-up four weeks after the experimental period, participants were asked to fill out a small battery of questionnaires (for details see electronic supplemental material). For the PC group, these were the only tasks.

For the AC and VR groups, as an additional task subjects were instructed to follow a lucid dreaming training schedule (see electronic supplementary material), which consisted of regularly asking themselves critically ‘Am I dreaming’ 5–10 times per day, particularly in situations that feel bizarre or dream-like. To convince themselves if they are indeed in a dream or not, they were asked to perform ‘reality checks' in these situations, which they logged using their phone. These logs showed that participants in both groups performed a virtually identical number of reality checks per day throughout training, namely 7.95 ± 1.65 in the AC group and 8.00 ± 1.20 in the VR group.

**Figure 1.**
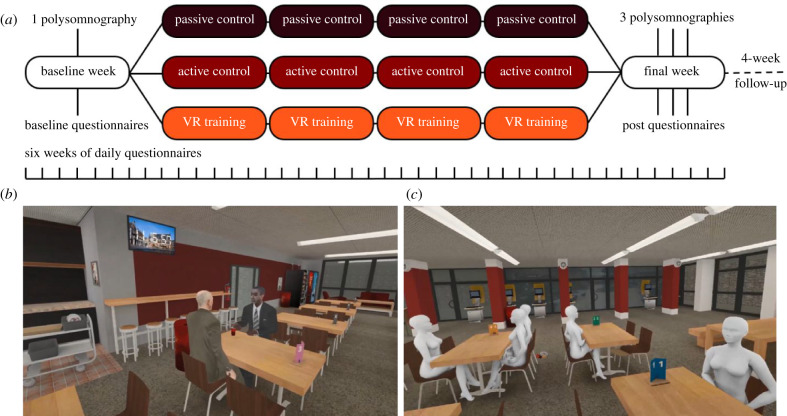
(*a*) Study design. After a baseline week including questionnaires and one night of polysomnography, participants were pseudo-randomized into one of three conditions. The experimental group followed a VR-enhanced training of lucid dreaming for four weeks, the active control group followed a simple lucid dreaming training for four weeks, whereas the passive control group received no training. In the sixth week, participants received three polysomnographies each in addition to questionnaires. Dream lucidity was measured each morning with the DLQ; after each polysomography with the LuCiD; and during nights with polysomnographies via eye signalling as verified by the EOG. (*b*,*c*) Screenshots of the custom-made VR training scenario ‘Spinoza Café'. (Online version in colour.)

The VR group in addition received 12 sessions of VR training (3 per week, 45 min each during daytime between 09.00 and 18.00 h), over four consecutive weeks. We used the HTC Vive head-mounted display with two ‘lighthouse' tracking beacons and the Steam VR client on Windows 7; PC components included an Intel Xeon E5-1620v2 CPU, EVGA GeForce GTX 1080 SC 8 GB GPU and 8 GB of 1600 Mhz DDR3 RAM, which were sufficient to consistently exceed 90 FPS on two simultaneous displays. The VR training exposed participants to a broad range of games, selecting specific games for re-play based on direct feedback. The overarching goal was to select games that felt as dream-like as possible, e.g. produced feelings of immersion into a virtual environment with (partly) surreal features while avoiding games that were fourth-wall breaking or difficult to engage with. Every early session (first two weeks) included the custom-made ‘Spinoza Café' scenario (figure 1) which involved performing the role of a waiter or waitress—delivering food to customers in the university canteen—which became progressively bizarre (clocks running backwards; customers suddenly staring at the participant or changing into mannequins; gravity failing) under control of the researcher. To increase the dream-like character, changes occurred outside of the field of the participants, i.e. they did not directly witness any changes, but only the result of the change when moving their gaze/attention to a different field of view. This produced an aesthetic which closely resembles dreaming, since alterations to working memory (among other cognitive changes) render otherwise intrusive, non-sequitur inconsistencies frequent and unremarkable from within the dreaming state. To keep participants engaged, later sessions focused on more difficult, visually diverse and complicated games (see electronic supplementary material).

### (b) Questionnaires

For the DLQ, only the items associated with the main *lucidity factor* were used for further analysis (see [9]). Likewise for the LuCiD, only items associated with the main dream *insight factor* were used (see [10]). Failure to recall a dream upon awakening was treated as ‘missing data' for the purpose of analysis, as were any aberrant cases such as parasomnia. In a single instance, a participant experienced a sleep paralysis event during the baseline week of recording and—not being aware of the phenomenon—erroneously described it as a ‘lucid’ dream. This data point was therefore excluded. For the DLQ, the mean of the first versus the final week was compared, whereas for the LuCiD, the one questionnaire of the first week versus the mean of the three questionnaires of the final week were compared. To test if incorporation of VR elements into dreams had an influence on the training effect, the mean of the daily question on VR-dream incorporation was correlated with the lucidity increase over training as measured by the DLQ or LuCiD. Whereas the densely sampled DLQ served as our primary outcome measure, the LuCiD served as an additional control measure to test for the robustness of results.

### (c) Statistical analysis

All statistical analyses were conducted in the R programming language (v. 3.5.1; [12]). Differences between the three groups were analysed using one-way ANOVA tests. Post-hoc pairwise comparisons were performed using the ‘emmeans' package [13], controlling for multiple comparisons using the Tukey test. Plots were inspired by the RainCloud plot [14].

### (d) Lucid dream eye signal verification analysis

Eye signal-verified lucid dreams were evaluated via SpiSOP software (RRID:SCR_015673, https://www.spisop.org) by two independent raters. Scaling for electroencephalography was 100 mV, 25 mV for electromyography and 100 mV for EOG.

## 3. Results

### (a) Questionnaires

**Figure 2.**
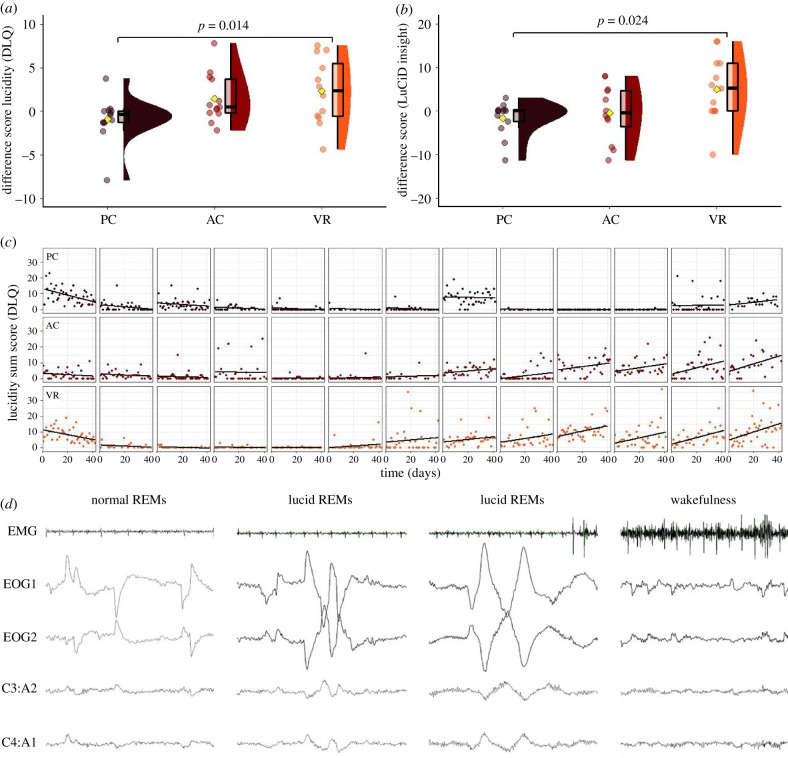
Average lucidity as measured by the DLQ (*a*) or LuCiD (*b*) post-training (final week), compared to individual baseline (first week). (*c*) Dream lucidity as measured by the DLQ over 42 days for all 39 participants, separated by group. Trend lines fitted to each time series using linear regression. (*d*) Successful eye signal-verified lucid dreaming in the final week by a participant from the VR group. (Online version in colour.)

See figure 2*c* for all raw DLQ scores over time. Group differences in the DLQ were not statistically significant in the average sum score of the first baseline week (*p* = 0.96, $\eta_{G}^{2}=0.003$). Groups, however, did differ in their DLQ sum score of the final week after training (corrected for individual baseline, i.e. mean DLQ scores of the first week), *F*_2,36_ = 4.48, *p* = 0.018, $\eta_{G}^{2}=0.199$ (figure 2*a*). A pairwise comparison revealed that the PC (*M* *=* −0.88) and VR (*M* *=* 2.94) groups differed significantly (*p* = 0.014), whereas the PC and AC (*M* = 1.47) groups did not differ (*p* = 0.177), nor did the VR and AC groups (*p* = 0.493).

Average baseline scores did not correlate with training increases in the VR group (*r* = 0.18, *p* = 0.56), the AC group (*r* = 0.48, *p* = 0.10) or both combined (*r* = 0.29, *p* = 0.15), i.e. training effects were largely independent from starting lucid dreaming skills. VR dream incorporation was not significantly associated with training success (*r* = 0.3, *p* = 0.16). We further checked for immediate effects of VR training on dream lucidity in the subsequent night by comparing DLQ sum scores after days which included VR training versus days without training, but found no effect: *t*_23.9_ = −0.113, *p* = 0.911, *d* = −0.044. In addition, the VR group did not significantly incorporate aspects of the VR sessions in their dreams more in the nights after training sessions compared to nights without preceding training sessions: *t*_19_ = −1.36, *p* = 0.19, *d* = −0.533. The DLQ results were supported by analysis of the LuCiD results which showed comparable group differences (*F*_2,35_ = 4.29, *p* = 0.022, $\eta_{G}^{2}=0.197$; figure 2*b* and electronic supplementary material); the pairwise comparison revealed that the PC (*M* = −1.62) and the VR (*M* = 4.99) groups differed significantly, *p* = 0.024. No group differences were found between the PC and the AC (*M* = −0.42; *p* = 0.88) nor the VR and the AC groups (*p* = 0.08).

### (b) Polysomnography

Twelve participants described experiencing lucid dreams over one or more nights while wearing polysomnography equipment, during the final week study (eight from VR, four from AC). Nobody from the PC group described experiencing a lucid dream during polysomnography. Additionally, four participants from the VR group reported experiencing lucid dreams over two consecutive nights, while a single participant from AC described experiencing a lucid dream during the baseline recording, bringing the total number of prospectively lucid recordings to 17. Of these, six participants reported eye signal verification attempts (four participants with a total of five dreams from VR, two participants from AC). Agreement between raters for clear eye signals was reached in three such cases, all from the VR group (see two examples from a single participant in figure 2*d*).

## 4. Discussion

The original rationale for this study was for the provision of synthetically generated dream-like environments and for participants to question their reality with these environments, in order to ascertain whether the success of lucid dreaming training could be improved. VR training led to statistically significant increases in dream lucidity compared to the passive control group, as evinced through both dream lucidity self-ratings in the DLQ and LuCiD questionnaires. Contrary to our hypothesis, we did not observe significant differences between the passive and active control conditions, nor between the active control condition and VR training.

Given our results, it is possible that the use of VR does not substantially increase classical lucid dreaming training. However, despite the lack of statistically significant differences in our primary outcome measures between VR and classical training (or classical training and a passive control condition), both behavioural and neurophysiological data support the assumption that the apparent gradient across the three training conditions might reflect actual differences: participants of both training groups performed a virtually identical amount of reality checks throughout training; however, twice as many participants of the VR group reported full-blown lucid dreams with or without eye signal verification attempts compared to the active control group, whereas none were reported from participants of the passive control condition. Strikingly, all three actually eye signal-verified lucid dreams occurred in the VR group after training. These observations are well in line with the assumption that interventions might have added cumulatively.

The failure to statistically back up a potential superiority of VR-enhanced training compared to lucid dreaming training without VR components, or of the latter to the passive control condition, might be interpreted as due to a lack of statistical power: even in trained (or naturally experienced) lucid dreamers, lucid dreaming is rare. While lucid dreaming is not a strictly dichotomous phenomenon and degrees of lucidity exist, the most plausible (and desirable) effect of any lucid dreaming training with reality testing is not in the generation of small lucidity increases, evenly distributed across all nights, but increased frequency of sporadic, full-blown lucid dreams. In line with this reasoning, instances of extremely high-scoring lucid dreams showed the strongest training effect (see electronic supplemental material, analyses). Either way, we can conclude that both pure lucid dreaming training as applied in our study, as well as its potential enhancement by VR, have only moderate effects, or work only in some participants. A closer look at the individual training data appears to support the latter interpretation (figure 2*c*).

While the use of VR tentatively appears effective for lucid dreaming training, the precise mechanism may not have been precisely that which the study was originally designed to test. Even though VR dream incorporation did not correlate significantly with increases in dream lucidity, anecdotal and written reports by study participants suggest that dream incorporation might actually have played a role in reports of dream lucidity. However, such incorporated content was frequently noticed as being ‘reality-like' within the context of the dream, thus reminding the participant of the research project and its aims as a precursor to becoming lucid. In this sense, the instantiation of associative memory traces conducive for lucidity may have instead been formed between the novel and aesthetically enriched experiences of VR and the knowledge that *one is taking part in a research project*. As such aesthetic content was gradually incorporated into participants' dreams, it stood a strong chance of being noticed, reminding them of the overarching goal within which the VR content had been provided.

Anecdotal participant reports pointed towards a third potential mechanism besides VR serving as a realistic dream environment and VR elements being incorporated into dreams: several participants within the VR group described mild to moderate feelings of dissociation following VR game exposure, which typically persisted for 1–2 h, and gradually diminished thereafter. Such dissociation symptoms are well known in the VR literature [15–17]: initially taken to be ‘vertigo' or ‘sea sickness' type effects, this phenomenon—referred to in the literature as ‘VRISE'—is quite distinct from motion sickness [18] and remains poorly understood. Descriptions of VRISE overlap with and quite accurately describe the dissociative symptoms exhibited by participants—a conclusion supported by the psychological literature, which independently describes VR technology as a potent tool for inducing dissociated states for clinical applications [19,20]. As we used VR hardware of the latest generation, these dissociogenic effects likely did not have their origins in the vestibular system as reported for older systems [21], but might be interpreted as a novelty effect given that our participants were VR novices. Potentially dissociogenic effects of VR, described in the literature and anecdotally reported in our study as well, are in line with previous descriptions of links between lucid dreaming and state dissociation [10,22–25], lucid dreaming and the prefrontal cortex [26–28]), and clinical dissociation and the prefrontal cortex [29,30]. Lucid dreaming has been shown to occur with stronger dream control in patients with bipolar disorders and schizophrenia ([31]; but see [32]), which include dissociative and depersonalization-like symptoms as part of their central pathology [33–35]. It could accordingly be argued that dissociative symptoms related to VR might instil a sense of ‘dissreality' and ‘reality scepticism' that increased the authenticity of reality checks. In other words: it could well have been a potential post-VR dissociative state that was ‘dream like', rather than the VR content itself, supporting the initial premise of the study, albeit through serendipitous and unforeseen secondary consequence of the primary intervention.

It therefore stands to reason that the tentative gains seen in the VR group could be explained through a combination of several overlapping factors: dream-like VR scenarios provided a training ground for metacognitive reflections; bizarre and novel VR content was subsequently incorporated into participants' dream imagery; and this reminded them of the study goal when noticed. It is further worth considering whether the VR experience itself could have exerted some dissociative effects, which postspectively provided a fertile and convincing (dream-like) psychological state from which to question one's reality, as part of the required lucid dreaming training. While the former of these was part of the original rationale of the study—albeit with slightly different memory associations in mind—the latter of these was entirely unforeseen. Since these interpretations of the potential mechanisms underlying the observed training effects remain speculative, future replications of this study would be well placed to differentiate between these potential explanations. For example, dream reports prompted after repeated awakenings could document potential dream incorporations of VR elements in more detail; experience sampling during the day might document the continuous presence of VR elements or potential dissociative experiences; administration of *state dissociation* questionnaires during baseline versus post-VR (e.g. [36]) and through detailed follow-up interviews with successfully lucid participants might identify, in greater resolution, how lucidity promoting memory traces were formed and experienced.

The need to augment, better control for and measure the success of lucid dreaming training via ‘reality' checks—in part, the original rationale of this study—was supported by considerable interindividual variability in participant feedback. Some participants retrospectively described the performance of their ‘reality’ checks in terms of a self-soothing, calming mindfulness—or in one case—an adaptation of an anxiety ameliorating exercise, learned during counselling. For other participants, the ‘reality' check constituted a form of ‘self-induced existential crisis' and a moment of ‘deliberate paranoia'. The somewhat uncomfortable nature of the *existential anxiety* that these cognitive processes can induce—arguably indispensable to the training effect—can easily be circumvented (in the form of poor compliance) without any way for the researchers to realize or correct for this. This could further explain the effectiveness of the VR training: from a dissociated state, the mandated ‘reality checks' serve to recruit the intended cognitive processes with much less margin for interpretation. It may therefore have been a matter of coincidence that, of the 5–10 reality checks required during any given day, some number of these were likely to have fallen within the 1–2+ hour dissociative window following VR training. Future attempts to replicate these findings would be well placed to consider this discussion point, and as much as possible, control for and attempt to measure the degrees of dissociation that follow VR exposure; the cognitive processes that underpin reality checks participant-to-participant; and the timing of such checks with regards to any induced dissociative windows. Furthermore, various improvements to VR software have been made since this study was first conceived. In particular, research technologies and software that specifically reproduce psychedelic-like alterations to the visual stream [37] may be of enormous benefit, particularly those which use machine learning and Bayesian inference to alter the visual stream in real time [38]. Moreover, expanding the use of VR to the provision of biofeedback training could also be of enormous benefit [39], and future studies of this nature would be well placed to consider expanding considerably beyond the recreational software market for the provision of VR training.

In conclusion, VR-assisted lucid dreaming training increased dream lucidity compared to a passive control condition. However, neither differences between VR training and classical lucid dreaming training as an active control condition, nor between classical lucid dreaming training and the passive control condition were significant, suggesting that training effects were moderate or only effective in some individuals. Beyond providing synthetic dream-like environments as a training ground for more genuine critical questioning of the reality of one's current phenomenal experience, mechanisms might involve incorporation of VR elements into dream imagery that served as mnemonic cues of the training situation, and lasting feelings of dissociation after VR sessions that further boosted ‘reality checks' as core elements of lucid dreaming training.

## Supplementary Material

Supplemental Material

## Data accessibility

Data, code and materials can be found in the electronic supplemental material and on https://osf.io/jrph2.
