# Supplementary material for: Virtual reality training of lucid dreaming
Source: Philos Trans R Soc Lond B Biol Sci. 2020 Dec 14;376(1817):20190697. doi: 10.1098/rstb.2019.0697 (PMC7741087; doi:10.1098/rstb.2019.0697)
Supplement: Supplemental Material [file rstb20190697supp1.pdf]

Virtual reality training of lucid dreaming – Supplemental Material

Jarrod Gott[1]*, Leonore Bovy[1]*, Emma Peters[1], Sofia Tzioridou[1,2], Stefano Meo[1], Çağatay Demirel[1], Mahdad Jafarzadeh Esfahani[1], Pedro Reis Oliveira[1], Thomas Houweling[3], Alessandro Orticoni[4], Anke Rademaker[1], Diede Booltink[1], Rathiga Varatheeswaran[5], Carmen van Hooijdonk[6], Mahmoud Chaabou[7], Anastasia Mangiaruga[8], Erik van den Berge[9], Frederik D. Weber[1], Simone Ritter[10], Martin Dresler[1]

[1]Donders Institute for Brain, Cognition and Behaviour, Radboud University Medical Centre, Nijmegen, The Netherlands

[2]Philipps University, Marburg, Germany

[3]University of Zurich, Zurich, Switzerland

[4]IRCCS San Raffaele Pisana, Rome, Italy

[5]Leibniz Institute for Resilience Research, Mainz, Germany

[6]School for Mental Health and Neuroscience, Maastricht University, Maastricht, The Netherlands

[7]Rivierduinen Institute for Mental Healthcare, Leiden, The Netherlands

[8]Technical University Berlin, Germany

[9]Department of Medical and Surgical Sciences, University of Bologna, Bologna, Italy

[10]Radboud University, Nijmegen, The Netherlands

[11]Institute for Management Research, Nijmegen School of Management, Radboud University, Nijmegen, The Netherlands

*equal contribution

Correspondence: Martin Dresler, martin.dresler@donders.ru.nl, https://dreslerlab.org

Donders Institute, Kapittelweg 29, 6525 EN Nijmegen, The Netherlands

Overview supplemental material

1. Questionnaires
2. Supplemental behavioural data
3. Lucid dreaming training
4. VR scenarios
5. Dream reports for eye signal-verified lucid dreams
6. Dream reports containing VR scenario incorporation
7. Supplemental discussion
8. Supplemental references
9. Further supplemental data: https://osf.io/jrph2

1. Questionnaires

The following tests and questionnaires were used within the study:

- Mannheim Dream Questionnaire (MADRE, Schredl et al., 2014)
- Dream Lucidity Questionnaire (DLQ, Stumbrys et al., 2013),
- Lucidity and Consciousness in Dreams Scale (LuCiD, Voss et al., 2013)
- Pittsburgh Sleep Quality Index (PSQI, Buysse et al., 1989)
- Reduced Morning-Eveningness Questionnaire (rMEQ, Adan & Almirall., 1991)
- Prospective and Retrospective Memory Questionnaire (PRMQ, Smith et al., 2000)
- Freiburg Mindfulness Inventory (FMI, Walach et al., 2006)
- Levenson Multidimensional Locus of Control Scales (LOC, Levenson, 1973)
- Positive and negative affect scale (PANAS, Watson et al., 1988)
- Beck Depression Inventory II (BDI, Beck et al., 1996)
- Visual Analogue Mood Scale (VAMS, van Rijsbergen et al., 2012)
- Alternative Uses Test (AUT, Guilford, 1967)
- Remote Association Test (RAT, Mednick, 1962; Chermahini et al., 2012)
- Visual Imagination Test (VIT, Skillicorn, 2013)
- Societal Problem Task (SPT, Maier, 1931; Duncker & Lees, 1945)
- Insight Problem Solving (IPT, Ritter et al., unpublished)

During screening, participants were asked for demographic data and their general lucid dreaming frequency in the form of the respective question of the MADRE. During the baseline week, participants filled out the MADRE to test for self-assessed (lucid) dream frequency of the past; PSQI for sleep quality; rMEQ for chronotype; FMI for trait mindfulness; LOC for trait locus of control; and PRMQ for trait prospective memory (self- or externally-cued). For research unrelated to our main study questions, further the PANAS, BDI, AUT, RAT, VIT, SPT, IPT were administered.

Over the 6 weeks of the study, participants day filled in a daily questionnaire comprising the VAMS (evening and morning), a brief dream report and the DLQ (morning); and for the VR condition and additional question “My dream was related to a virtual reality environment I have experienced” with a 6-point Likert scale (morning). The VAMS was collected for a different study, and thus not analysed.

During the final week, participants finished the same questionnaires/tasks as in the first week, however without demographic data, PSQI, rMEQ, PRMQ.

During a 4-week follow-up, participants filled out the PSQI to evaluate any effects of training on sleep quality; further the MADRE, FMI, LOC, PANAS, BDI. The PANAS and BDI were collected for a different study and thus not analysed.

2. Supplemental behavioural data

Lucid dreaming training: explorative analyses

Consistent with the DLQ results, we did not observe a significant relation between the average VR dream incorporation over the 4 training weeks increases in the LuCiD *insight* scores, r = 0.33, p = .138. Given major inconsistencies in MADRE scores of several participants due to technical issues or poor compliance, we could not test for differences in this rather coarse measure of lucid dream frequency over more extended periods.

We performed several explorative tests to elucidate potential factors influencing the training effect. Testing if trait mindfulness (potentially influencing the attention payed to the current state of mind) or self- or externally-cued prospective memory (potentially influencing the intention to recognize upcoming dreams as such), we did not observe any significant correlation between these parameters and lucid dreaming increases, either for both training groups separately or combined (see Supplemental Figure S3).

Testing if lucid dreaming training had an effect on sleep quality, mindfulness or internal locus of control, we did not observe any significant interactions between time and group for the PSQI ($F(2,68) = 0.88$, $p = 0.42$), the FMI ($F(4,105) = 0.05$, $p = 0.99$), or the LOC ($F(4,105) = 0.37$, $p = 0.83$).

To test if training increased particularly sporadic full-blown lucid dreams rather than a general level of lucidity in every night, we counted the amount of dream reports whose DLQ score exceeded 1, 2, 3, or 4 standard deviations beyond the individual mean of the baseline week. We found significant differences only for DLQ scores exceeding 4 SD of the baseline: $F(2,36) = 5.481$, $p = .008$, $\eta^2_G = .233$; see supplemental figure S4).

Reality Checks

To test if the training groups quantitatively differed in the intensity of their reality testing, in particular if the additional VR sessions increased the number of reality checks, we compared the daily logged reality check counts between training groups. We did not observe any differences between groups on the averaged total amount of checks, with VR participants performing 8.00±1.2 and active control participants 7.95±1.65 reality checks pay day; $t(20) = -0.0813$, $p = 0.936$.

Questionnaires

For questionnaire data at different time points not presented in the main manuscript, see Supplemental Table S1.

	passive control	active control	virtual reality
rMEQ	13.4 ± 1.16	15.1 ± 1.35	12.2 ± 0.82
PRMQ PSS	2.85 ± 0.18	2.92 ± 0.23	3.35 ± 0.16
PRMQ PSE	2.42 ± 0.24	2.77 ± 0.22	3.38 ± 0.19
PSQI, baseline week	5.0 ± 0.56	5.0 ± 0.38	5.0 ± 0.48
PSQI, follow-up	5.17 ± 0.47	4.46 ± 0.67	5.92 ± 0.69
FMI, baseline week	35.5 ± 1.41	39.0 ± 1.77	36.5 ± 2.17
FMI, final week	33.7 ± 1.77	37.5 ± 1.94	35.5 ± 2.36
FMI, follow-up	38.4 ± 2.17	38.4 ± 1.82	37.2 ± 1.86
LOC, baseline week	33.0 ± 1.91	32.8 ± 1.96	34.1 ± 1.48
LOC, final week	31.8 ± 1.79	34.5 ± 2.36	34.2 ± 1.67
LOC, follow-up	32.5 ± 2.32	36.5 ± 1.43	34.8 ± 1.01

Supplemental Table S1: Questionnaire data. rMEQ = reduced Morningness Eveningness Questionnaire. PSQI = Pittsburg Sleep Quality Index. PRMQ = Prospective and Retrospective Memory Questionnaire. PSS = Prospective short-term self-cued. PSE = Prospective short-term environmentally-cued. FMRI = Freiburg Mindfulness Inventory. LOC = Levenson Multidimensional Locus of Control Scale, internal locus of control.

Supplemental Figure S1: Association between baseline lucid dreaming as measured by the DLQ and training increases (see main manuscript).

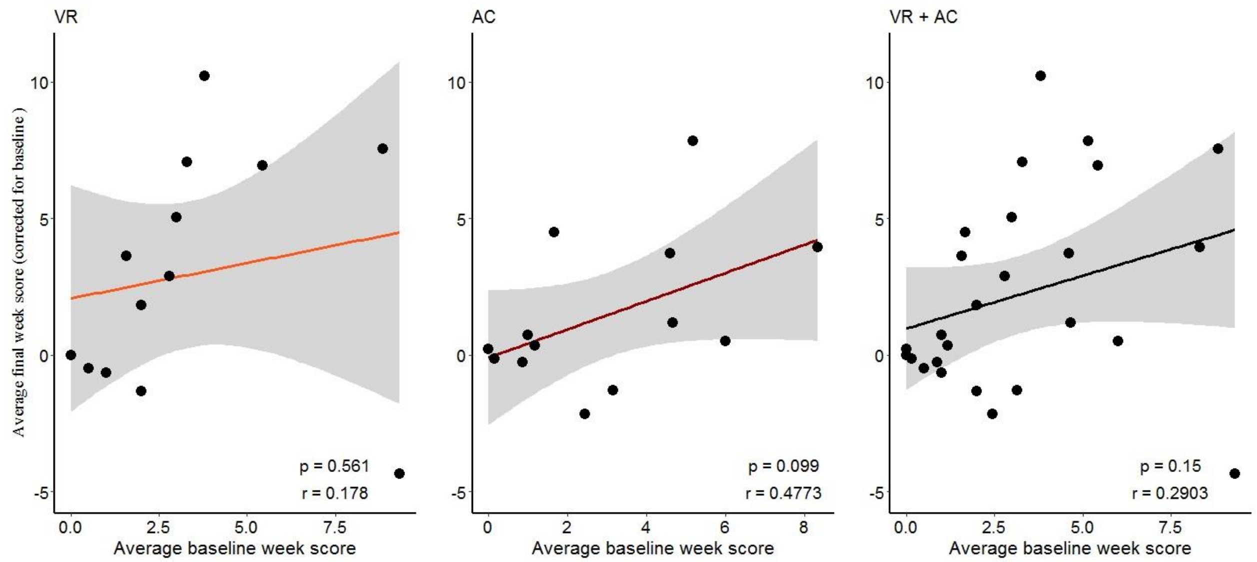

Supplemental Figure S2: Association between dream incorporation of VR elements and lucid dreaming increases as measure by the DLQ or LuCiD (see main manuscript).

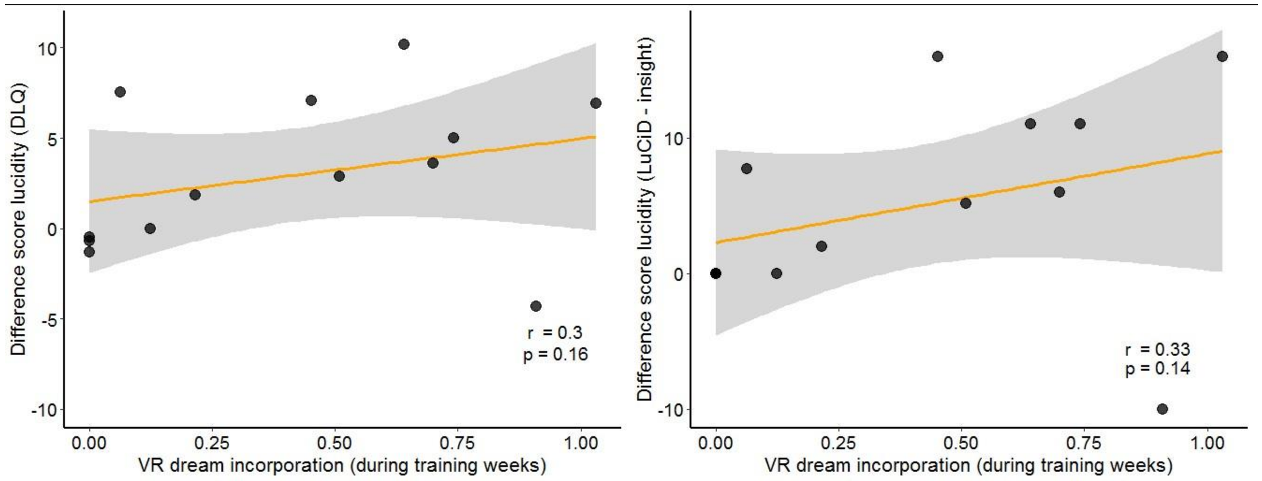

Supplemental Figure S3 (next page): Mindfulness, internal locus of control, prospective memory as potential predictors of training success. Neither the mindfulness (as measured by the FMI), nor internal locus of control (as measure by the LOC), nor self- (PSS) or environmentally-cued (PSE) short-term prospective memory significantly predicted lucid dreaming training gains as measured by the DLQ.

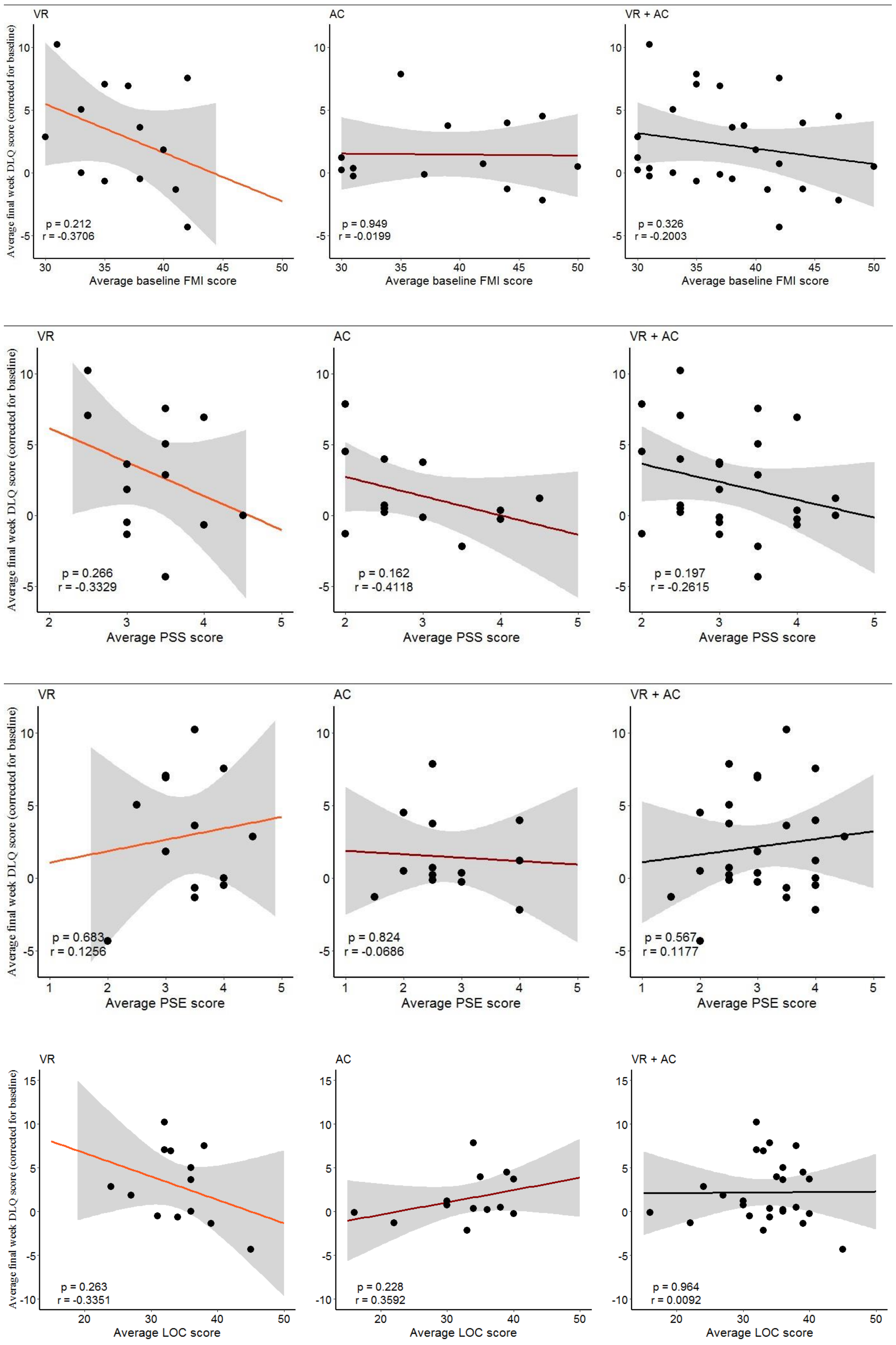
VR
AC
VR + AC
Average final week DLQ score (corrected for baseline)
p = 0.212
r = -0.3706
p = 0.949
r = -0.0199
p = 0.326
r = -0.2003
Average baseline FMI score
p = 0.266
r = -0.3329
p = 0.162
r = -0.4118
p = 0.197
r = -0.2615
Average PSS score
p = 0.683
r = 0.1256
p = 0.824
r = -0.0686
p = 0.567
r = 0.1177
Average PSE score
p = 0.263
r = -0.3351
p = 0.228
r = 0.3592
p = 0.964
r = 0.0092
Average LOC score

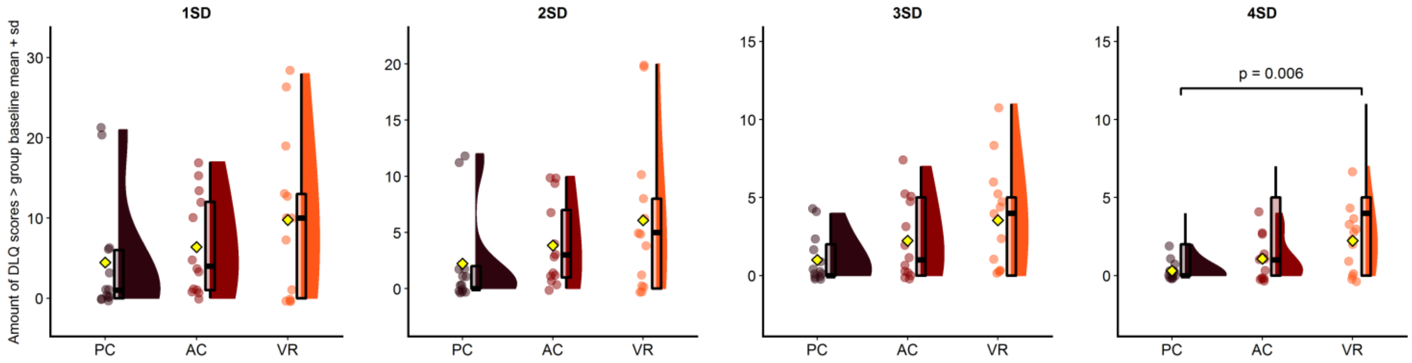

Supplemental Figure S4: Analysis of training effects on full-blown lucid dreams as measured by extreme DLQ scores. Tested are counts of lucid dream reports whose DLQ score exceeds 1, 2, 3, or 4 SD beyond the individual baseline (i.e. the first week). The most extreme DLQ values show the strongest training effect.

3. Lucid dreaming training

Participants of both the VR training group and the active control group received the same instructions for daily lucid dreaming training. In an individual introduction session, they were introduced into the concept of critically questioning their current state of mind to increase awareness of the discrepancies between real-life events and the strange events of dreams, thus serving as a trigger for achieving lucidity.

Specifically, they were instructed to ask themselves 5-10 times a day “Am I dreaming or not?”, particularly in situation that subjectively felt unusual, dream-like, or strongly emotional. Participants of the VR training group were asked to do this in particular during VR sessions. All participants were further instructed not to answer this question mindlessly, but rather try to convince themselves that they are in a dream, and to look around for any strange things or inconsistencies that might be indicative of a dream. In addition, they were asked to perform “reality checks” to reliably test their current state of mind, for example trying to remember what happened just a few seconds ago. They received a “probe card” with the printed question “Is this a dream?”, which they were instructed to observe closely for any inconsistencies in spelling or style; and look on the backside and frontside again to check if anything changes during the process. They were further instructed to choose specific times/occasions such as “while sitting at breakfast” or “while riding the bus to work” serving as regular cues for critical questions, and to log their reality checks using their smartphone after performing them. Throughout the experimental periods, participants received daily reminders via email, and personal reminders when they missed logging.

In addition, participants were instructed to go to sleep with the intention that upcoming dreams will be lucid; to carry out a particular action while dreaming (e.g. try to fly); and to move their eyes left-right-left-right when realizing to be in a lucid dream, try to move your eyes left-right-left-right.

Finally, participants of the training group were provided with written information and instructions on the subject of lucid dreaming, taken from the book “Exploring the world of lucid dreaming” (LaBerge & Rheingold, 1991). This specifically included the sub-sections ‘*Introduction to Lucid Dreaming, Dream-signs: Doors to Lucidity, The Dream-sign Inventory, Lucid Dreaming Induction Training*’ in addition to an additional section titled ‘Instructions for you’ written explicitly for the participants.

4. VR scenarios

Our starting and mainly used VR scenario was the custom made ***Spinoza Café***, which was developed on the basis of an earlier version of a virtual replica of the university cafeteria (Ritter et al., 2012), hence a recognizable place for our student participants. Participants were tasked with serving customers their food and drinks, and cleaning up afterwards. Although the cafeteria itself is quite large, we opted to section off a space with only 4 tables, so our participants can reach everything within walking distance of the 5*5m^2 VR lab space. The scenario allowed researchers to manually or automatically spawn in customers. Once seated, participants used Vive controllers to pick up customers' orders at the counter and deliver it to the right table. Once the customers are finished, they left behind an empty cup and/or plate that had to be cleaned up before a new customer could sit in that spot. Empty cups had to be thrown in a bin, plates and trays had to be put on a little cart. Meanwhile, researchers could trigger different dream-like events, with an increasing level of surreality: change posters on the wall; change the clock on the wall to either fast forward or rewind; shuffle table numbers; move the trash bin (to different spots, but also inverted on the ceiling); male all customers stare at the participant; change all customers into mannequins; turn off gravity. All events happened behind the back of the participant to prevent jarring pop-in effects and increase the dream-like quality of the experience.

To keep the training engaging and ensure that participants had highly variable experiences just as during actuals dreaming, the following VR scenarios were used in addition.

Accounting, Crows Crows Crows, Berlin (Germany)
A surreal puzzle game with dark humour and bizarre aesthetics. Frequently used.

Blueshift, Zulubo Productions, https://www.zulubo.com
An action game based on flying/flight physics. Difficult. Rarely used.

Cosmic Wandering, Punchey, https://punchey.itch.io
Fantasy/puzzle game in dream like environment. Difficult. Rarely used.

Found, FoundVR, San Francisco (United States of America)
Puzzle/scenery game in dream like environment. Easy and aesthetically engaging. Frequently used.

Longbow (The Lab), Valve Corporation, Washington (United States of America)
Action game based on archery skills. Progressively difficult over consecutive levels. Concentration/dexterity exhausting. Popular and frequently used.

Outside the Box, Megasteakman, https://megasteakman.itch.io
Surreal puzzle game with metacognitive attributes. Very short, little replay value. Typically played once or twice per participant.

Quanero, Laserboys3000
Surreal/dream-like puzzle game, set on a space station, involving time travel. Too difficult for some participants, but highly popular with others. Used case-by-case.

Senza Peso, Kite & Lightning, Los Angeles (United States of America)
An ‘animated opera’ in a dream-like environment with no player involvement. Enjoyable for some participants but boring for others. Used case-by-case.

SightLineVR, Solirax, Prague (Czech Republic)

A dream-like scenery-based game, with minimal puzzle components. Enjoyable and frequently used.

Slingshot (The Lab), Valve Corporation, Washington (United States of America)
Action game in an industrial environment. Rarely used—predominantly as an alternative to ‘Longbow’ to add variety.

Spirit Realm, Magic House Creative
Dream-like and psychedelic-like scenery game, with no player involvement. Used moderately until participants report boredom.

The Cubicle, Pelican Party Studio, Rotterdam (Netherlands)
Puzzle/scenery game in dream-like environment, with moderate player involvement. Popular and used frequently.

The Gallery: Starseed, Cloudhead Games ltd., Brits-Columbia (Canada)
Difficult puzzle game in desolate/dissociative environments. Popular with some participants but too difficult for others. Used case-by-case.

The Price of Freedom, Construct Studio Inc., San Francisco (United States of America)
Complex puzzle game with cold war/film noir themes. Generally popular and used frequently.

The Rose and I, Penrose Studios, San Francisco (United States of America)
Animated film with no player involvement. Short and used frequently.

Velocity, Zulubo Productions, https://www.zulubo.com
Flying/physics game in dream-like environment. Very popular and used frequently.

Waltz the Wizard, Aldin Dynamics, Reykjavik (Iceland)
Puzzle/action game in nightmare-like environment. Used frequently in early sessions, and case-by-case in later ones.

Please note that the use frequency of the different VR scenarios was not systematically assessed, but only estimated post-hoc.

5. Dream reports for signal-verified lucid dreams

Report 1

Report sent via official project email address on the following morning. Verbatim: (*"Hey, I will bring the EEG at 11:30, I actually had a lucid dream! I hope I managed to move my eyes right but I think I did."*).

Dream transcript: (*"I was in the gym and learned self-defence. There was a huge giant I couldn't fight → dream became lucid: I told myself he isn't real and that's how I realised I was dreaming"*)

2 pairs of eye signals

DLQ scores [1–12]

[3 3 3 3 2 3 3 2 2 3 3 3]

LuCiD scores

Insight—27, Realism—8, Control—19, Memory—12, Thought—11, Dissociation—4

Report 2

Dream transcript: (*"I had a few dreams but they got kind of blurred and mixed with each other when I woke up. One of the dreams was lucid and I remember it well. I was walking up to the hill I knew from my childhood – it looked different, bigger and more majestic. The sun was going down and I felt almost euphoric seeing all this beauty. All of a sudden it got really windy. I saw a tornado coming at me. I started running, but another tornado blocked my way out – I was stuck. I started to panic and breathe heavily, trying to think of an escape and then I realized this is way too much stress for a normal life situation. The tornados started to slow down and my breath started to slow down when I realized I'm in a dream too. I tried to move my eyes left and right but I'm not sure if I did it still in a dream or being half-conscious."*)

1 pair of eye signals

DLQ Scores [1–12]

[3 2 0 2 3 1 1 0 3 2 3 2]

LuCiD scores

Insight—19, Realism—9, Control—12, Memory—6, Thought—10, Dissociation—5

Report 3

Dream transcript: (*“I was playing a game on my phone. I was really inside the game, which kind of follow the rules of the actual game, but there were some additional options/ features and I won the game. I woke up in my bed (with the EEG, etc. in place) in my dream I was awake in bed for a while and I couldn’t fall back asleep. I thus decided to go down to the bathroom to pee and check the electrode on my chin to see if it was still attached (it was a bit loose). Once I was looking in the mirror. I was debating whether or not I should reapply it, so I checked the time to see how much time was left but the time didn’t make sense, so I looked away and back again a couple of times. It kept changing so I realized I was dreaming. I got excited and thought about what I could do. I remembered I had the EEG on and that I should try to move my eyes. I did that, but felt the same as last time and didn’t want to wake myself up again so I stopped before I woke up again and thought about what else I could do. I thought about flying but it felt like I couldn’t, as I was in my tiny bathroom. I wanted to step through the door, but remembered I could do anything, so I tried to go through the wall instead. That didn’t go to smoothly, as everything started shaking and vibrating, but I managed to push through. I was then in my bedroom and knocked over the laundry and got kind of stuck in it. I then rose up from the mess and looked down at the scattered laundry. I was exhausted from the effort, and didn’t really know what else to do so, I went back to bed, where I was actually laying. In my dream I fell back asleep and woke up from deep voices that sounded really close by (as if men were talking to each other right under our bed). I wasn’t sure if I was still dreaming or this was real, so I tried to wake up my boyfriend to ask him, but he wasn’t waking up. It felt really threatening.”*)

1 pair of eye signals

DLQ Scores [1–12]

[3 1 1 3 2 2 4 0 3 1 3 4]

LuCiD scores

Insight—19, Realism—13, Control—18, Memory—13, Thought—11, Dissociation—0

6. Dream reports containing VR scenario incorporation

Note: Identifiable information such as names and places have been changed

Report 4

Dream transcript: (*"I was at one of my high school friend's house in Singapore, and we are planning to go to the beach. However, my mom did not allow me since I am very badly sunburned. But since it's a hot day, I wore a swimming suit anyway. My friends all complained that I am not going but they left anyways. Feeling bad, I snuck out of the house and went to the beach, where they had a plane-flying competition. Somehow, someone nominated me and so there I went on one of the planes. The plane and flying mechanism reminded me of the VR game where you pretend to be a robot* and just fly around with wings. I was a very good pilot and so I won. But during flying I felt like the other pilot was also me, and I got very confused because I can see myself in 2 places. This confusion made me land my plane early to see the face of the other pilot. But the face was a blur. Then time got fast forwarded and me in a beach hut with my mom. She wanted to buy a beach house so we went looking for a beach house. She had contact with an old guy who gives advice about property. The old guy's house had no floor, but it was all plastic and I hated his house and so I left."*)

Game Incorporated: "Velocity"

Dream Incorporation score: 3

DLQ scores [1–12]

[2 0 2 2 2 2 1 1 3 2 2 0]

Report 5

Dream transcript: (*"I thought it was not a dream, because everything felt so rea. I was in my room as usual, when I heard my morning phone alarm. Usually, its behind my head on the shelves, but this time it was far away on the study desk. I thought "well this is weird" but I stood up and proceeded to silence it. When I did, sparkling yellow lights started flying around, just like in the VR*. Then I said: "This is a dream". The sky started changing colors and then I realised it was s dream. I went out into the hallway and there was nobody, which is really rare especially in student housing. The hallway was dark and lights flickered through the fridge light. I ended up walking around for sometime and got bored and went to my bed to lie down again.")*

Game Incorporated: "Waltz of the Wizard"

Dream Incorporation score: 2

DLQ scores [1–12]

[3 2 1 3 3 2 4 0 4 2 3 1 2]

Report 6

Dream transcript: (*"One virtual reality scene occurred: The garden that I needed to explore with the castles.* It was very harmonic and I was with my friend on a beautiful summer day. We were laying on the blanket on the grass talking. A good friend of mine whose name is Maria read one of my dream diary entries (which does not really exist) but she was not supposed to read it because it was about her and intimate. She talked to me about it (more like confronted me) and I was very sad but at the same time very happy about what she said to me, emphasizing the beauty of our friendship."*)

Game Incorporated: "Destinations"

Dream Incorporation score: 4

DLQ scores [1–12]

[2 1 0 0 2 0 3 0 0 1 0 0]

Report 7

Dream transcript: (*"I dreamed a lot about the presentation I will hold next week. I was part of a group which was wandering around in nature. I was climbing, I dreamed that I put on VR glasses and was then in a different world from which I had to escape."*)

Game Incorporated: VR System

Dream Incorporation score: 3

DLQ scores [1–12]

[1 1 2 2 1 2 3 2 3 2 1 1]

Report 8

Dream transcript: (*"There was a procedure to somehow categorize people (dystopian vibes) and I was somehow assigning numbers to people. My boyfriend and I went to an animal shelter. There was the same setting as one VR game.."*)

Game Incorporated: ???

Dream Incorporation score: 2

DLQ scores [1–12]

[1 1 1 1 1 1 2 1 1 1 1 1]

Report 9

Dream transcript: (*"I only remember that it was about X'mas, and I was thinking: "I've dreamed about things related to the reality recently. I only remember that I was looking my hand as what I did during VR training,* and I thought "Oh! Finally I have applied VR in the dream!". Afterwards, the stories changed fast and I was always trying to figure out clearly about the current scene. Sometimes I know my body's position in reality but sometimes not.)*

Game Incorporated: VR System

Dream Incorporation score: 3

DLQ scores [1–12]

[3 2 2 2 2 1 3 1 1 0 2 2]

Report 10

Dream transcript: (*"1st dream: I was sitting in the Spinoza cafe with my friends talking. 2nd: I was walking through the long corridors and couldn't find the way out. 3rd: me and my friend were running to win a bet and while we were running he changed into 3 different people."*)

Game Incorporated: "Spinoza Café"

Dream Incorporation score: 2

DLQ scores [1–12]

[1 0 2 1 1 0 2 0 3 0 2 0]

Report 11

Dream transcript: (*"I was a witch, and I realized I had a teenage daughter I have to save from witch conversion school lead by people afraid of dark magic. To do that, I entered a video game (because that was the only way to get to the school). I was flying and trying to escape all the people who wanted to kill me. I got to an old castle* and instead of my daughter I found a very old and tired witch. When I tried to help her get out of the castle, because of protection spells she burned alive in front of me and her body turned dust. Then I realized I was dreaming but it was a dream within a dream, where I was still a witch and sleeping in a bed with my ex. I closed my eyes and got back to the other dream to finally find my daughter. When I saw her by the window I tried to get to her, but there were too many spells protecting the house and I couldn't."*)

Game Incorporated: "Waltz of the Wizard"

Dream Incorporation score: 3

DLQ scores [1–12]

[2 2 1 3 1 2 0 0 4 0 1 1]

Report 12

Dream transcript: (*"I missed the EEG and had to make many stupid explanations. It all started from me seeing just colours around me* and I thought that "in real life there are not as beautiful."*)

Game Incorporated: "Waltz of the Wizard"

Dream Incorporation score: 2

DLQ scores [1–12]

[1 1 2 2 1 1 0 2 1 1 1 1]

Report 13

Dream transcript: (*"It was some mixture of Starseed* (the VR-game) and a German time-travel movie I watched. The environment looked like the laboratory in the game and I'm pretty sure I travelled through time."*)

Game Incorporated: "Starseed"

Dream Incorporation score: 4

DLQ scores [1–12]

[0 0 1 0 1 0 2 0 4 0 0]

Report 14

Dream transcript: (*"I was at some scene, outside in a small town square or something like that. Something bad had just happened which I had seen. I don't remember what exactly, but some kind of bomb went off, or people were shotting at a crowd. Once it had happened, I knew I had to go back (both physically and temporally)* to warn the people at the square that the bad thing was about to happen and to save them. Time was reversed, but I could move (freely) through it. I warned the people, but I wasn't able to save them all (just some). Together with the people that I did manage to warn and that actually listened, I was running away from the danger. I dropped my phone at some point, cracking the screen. My boyfriend was there and I told him that now both our screens were cracked. We also went on a plane at some point, but the pilot was not well maybe under the influence of alcohol or drugs, so she was trying to take off, but we were going left and right and almost hitting trees. By thinking, no, we have to be save, this is not happening, the plane somehow swirled back on course and landed safely. I also at some point developed a rash as an allergic reaction to my night cream that I had put on."*)

Game Incorporated: "Quanero"

Dream Incorporation score: 3

DLQ scores [1–12]

[1 0 0 2 1 2 2 0 1 2 1 1]

7. Supplemental discussion

A confounding factor that should be noted is an apparent bias resulting from 'enthusiasm' drift, which might have masked actually stronger increases in the two training groups. Whereas we would have expected an increase in lucid dreaming even in the passive control group just by paying attention to this phenomenon on a daily basis, DLQ scores declined in 11 of 13 participants of the PC group. The most plausible explanation we could arrive at to explain these decreases was the presence of remarkable enthusiasm and optimism relating to the aims of the study (becoming more lucid) which inflated lucidity scores in the initial weeks; perhaps through an understandable combination of positive and wishful thinking on the part of study participants. The tapering-off in DLQ scores could have therefore been a gradual regression towards an 'accurately' reportable baseline, as participants became fatigued, disenchanted and generally despondent that the training would produce results. Needless to say, this implies that VR and AC groups may have been similarly effected; with measures training increases consisting of those which stood out above such refractory deflation. It could alternatively have been the case that the successful VR and AC participants had their biases further fuelled; with self-exaggeration of scores being procedurally encouraged as training milestones were reached.

These considerations were explored and tested at the end phase of this project; supporting many of our intuitions about the data collected. A number of participants were re-contacted some weeks after concluding their involvement, and had their own DLQ dream diary entries stripped of identifying temporal and other information, randomised in order, and sent back; with instructions to re-fill the quantitative numerical components based on their written and personal recollection of the dream. This produced strong correlations with the original reports (Cronbach's Alpha = 0.43, 0.465, 0.633, 0.948; $p = 0.04, 0.02, 0.002, <0.0001$ respectively) justifying—at least in principle—the mnemonic validity and accuracy of retrospective dream lucidity reappraisal. Most importantly, this provided a relatively bias-free re-examination of participant' impressions of their lucidity, controlling for variation in mood, enthusiasm and optimism over the 42 days. One VR participant whose data reported a severe negative trend, upon re-analysis, saw remarkable gains (see Supplemental Figure S4); an observation backed up by the participant's own anecdotal reports of having "significantly more" lucid dreams as result of the intervention. Additionally, the PC participant whose data showed the most severe decline, upon re-evaluation, produced a near-uniform flat gradient—as one would expect from a passive control condition. Without comprehensive re-evaluation on a larger sample size, further conclusions are difficult to draw; however it does appear that longitudinal data collected on a highly subjective, fundamentally oscillatory/noisy quanta such as dream lucidity is apt to be overtaken by larger shifts in participants affect, at least in certain individuals. Future studies would do well to control for this possibility.

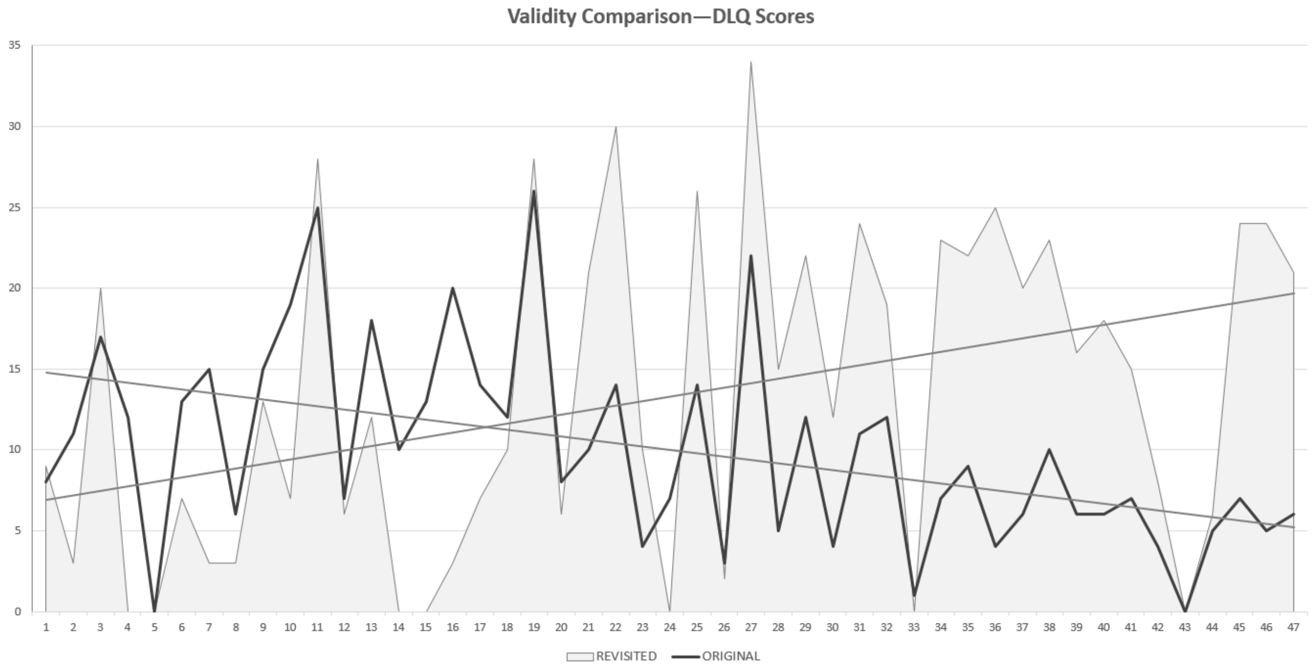

Supplemental Figure S4: Validity comparison between *originally* and *postspectively revisited* (order randomised, completed on a single sitting) DLQ questionnaires. Despite showing opposite trend-lines, both sets correlate considerably (Cronbach's Alpha = 0.465, p = 0.018). When the bias was corrected for through subtraction of linear estimations, both sets correlated significantly more (Cronbach's Alpha = 0.834, p < 0.001) indicating highly accurate lucidity estimations from postspective dream diary analysis; compensating for longitudinal affective drift. In this case from the VR training group, a potentially positive trend over training – consistent with the retrospective appraisal by the participant – might have been masked by some sort of drift, resulting in a seemingly negative trend over the course of the study.

8. Supplemental references

1 – Schredl, M., Berres, S., Klingauf, A., Schellhaas, S., & Göritz, A. (2014). The Mannheim Dream questionnaire (MADRE): Retest reliability, age and gender effects. International Journal of Dream Research, 7(2). 141-147

2 – Stumbrys, T., Erlacher, D., Schredl, M. (2013). Testing the involvement of the prefrontal cortex in lucid dreaming: a tDCS study. Consciousness and Cognition, 22: 1214-1222.

3 – Voss, U., Schermelleh-Engel, K., Windt, J., Frenzel, C., & Hobson, A. (2013). Measuring consciousness in dreams: the lucidity and consciousness in dreams scale. Consciousness and Cognition, 22(1), 8-21.

4 – Buysse, D.J., Reynolds, C.F., Monk, T.H., Berman, S.R., & Kupfer, D.J. (1989). The Pittsburgh Sleep Quality Index: a new instrument for psychiatric practice and research. Psychiatry res, 28(2), 193-213.

5 – Adan, A., & Almirall, H. (1991). Horne & Östberg morningness-eveningness questionnaire: A reduced scale. Personality and Individual differences, 12(3), 241-253.

6 – Smith, G., Del Sala, S., Logie, R.H., & Maylor, E.A. (2000). Prospective and retrospective memory in normal ageing and dementia: A questionnaire study. Memory, 8(5), 311-321.

7 – Walach, H., Buchheld, N., Buttenmüller, V., Kleinknecht, N., & Schmidt, S. (2006). Measuring mindfulness—the Freiburg mindfulness inventory (FMI). Personality and individual differences, 40(8), 1543-1555.

8 – Levenson, H. (1973). Multidimensional locus of control in psychiatric patients. Journal of consulting and clinical psychology, 41(3), 397.

9 – Watson, D., Clark, L.A., & Tellegen, A. (1988). Development and validation of brief measures of positive and negative affect: the PANAS scales. Journal of personality and social psychology, 54(6), 1063.

10 – Beck, A.T., Steer, R.A., & Brown, G.K. (1996). Beck depression inventory-II. San Antonio, 78(2), 490-498.

11 – van Rijsbergen, G.D., Bockting, C.L., Berking, M., Koeter, M.W., & Schene, A.H. (2012). Can a one-item mood scale do the trick? Predicting relapse over 5.5-years in recurrent depression. PloS one, 7(10).

12 – Guilford, J.P. (1967). Creativity: yesterday, today and tomorrow. Journal of Creative Behavior, 1(1), 3-14.

13 – Mednick, S. (1962). The associative basis of the creative process. Psychological review, 69(3), 220. https://doi.org/10.1037/h0048850

14 – Chermahini, S. A., Hickendorff, M., & Hommel, B. (2012). Development and validity of a Dutch version of the Remote Associates Task: An item-response theory approach. Thinking Skills and Creativity, 7(3), 177-186. https://doi.org/10.1016/j.tsc.2012.02.003

15 – Skillicorn, N. (2013). 30 days of creativity training. London: Improvised.

16 – Maier, N.R. (1931). Reasoning in humans. II. The solution of a problem and its appearance in consciousness. Journal of comparative Psychology, 12(2), 181.

17 – Duncker, K., & Lees, L.S. (1945). On problem solving. Psychological monographs, 58(5), i.

18 – Ritter S., Damian, R.I., Simonton, D.K., van Baaren, R.B., Strick, M., Derks, J., Dijksterhuis, A. (2012). Diversifying experiences enhance cognitive flexibility. Journal of Experimental Social Psychology 48, 961-964

19 – LaBerge, S., & Rheingold, H. (1991). Exploring the world of lucid dreaming. New York: Ballantine Books.

9. Further supplemental data

Further supplemental data and analysis scripts can be found on https://osf.io/jrph2
